# Ethnic differences in Internal Medicine referrals and diagnosis in the Netherlands

**DOI:** 10.1186/1471-2458-8-287

**Published:** 2008-08-14

**Authors:** Loes C Lanting, Aart H Bootsma, Steven WJ Lamberts, Johan P Mackenbach, Inez MA Joung

**Affiliations:** 1 Erasmus MC, Department of Public Health, Rotterdam, The Netherlands; 2 Erasmus MC, Department of Internal Medicine, Rotterdam, The Netherlands

## Abstract

**Background:**

As in other Western countries, the number of immigrants in the Netherlands is growing rapidly. In 1980 non-western immigrants constituted about 3% of the population, in 1990 it was 6% and currently it is more than 10%. Nearly half of the migrant population lives in the four major cities. In the municipality of Rotterdam 34% of the inhabitants are migrants. Health policy is based on the ideal that all inhabitants should have equal access to health care and this requires an efficient planning of health care resources, like staff and required time per patient. The aim of this study is to examine ethnic differences in the use of internal medicine outpatient care, specifically to examine ethnic differences in the reason for referral and diagnosis.

**Methods:**

We conducted a study with an open cohort design. We registered the ethnicity, sex, age, referral reasons, diagnosis and living area of all new patients that visited the internal medicine outpatient clinic of the Erasmus Medical Centre in Rotterdam (Erasmus MC) for one year (March 2002–2003). Additionally, we coded referrals according to the International Classification of Primary Care (ICPC) and categorised diagnosis according to the Diagnosis Treatment Combination (DTC). We analysed data by using Poisson regression and logistic regression.

**Results:**

All ethnic minority groups (Surinam, Turkish, Moroccan, Antillean/Aruban and Cape Verdean immigrants) living in Rotterdam municipality, make significantly more use of the outpatient clinic than native Dutch people (relative risk versus native Dutch people was 1.83, 1.97, 1.79, 1.65 and 1.88, respectively).

Immigrant patients are more likely to be referred for analysis and treatment of 'gastro-intestinal signs & symptoms' and were less often referred for 'indefinite, general signs'. Ethnic minorities were more frequently diagnosed with 'Liver diseases', and less often with 'Analysis without diagnosis'. The increased use of the outpatient facilities seems to be restricted to first-generation immigrants, and is mainly based on a higher risk of being referred with 'gastro-intestinal signs & symptoms'.

**Conclusion:**

These findings demonstrate substantial ethnic differences in the use of the outpatient care facilities. Ethnic differences may decrease in the future when the proportion of first-generation immigrants decreases. The increased use of outpatient health care seems to be related to ethnic background and the generation of the immigrants rather than to socio-economic status. Further study is needed to establish this.

## Background

Health policy is based on the ideal that all inhabitants should have equal access to health care. US studies have found ethnic differences in the use of health care with lower consumption rates for people from ethnic minorities [1,2]. European studies on ethnic differences in the use of health care have reported mixed outcomes. Some studies reported higher rates by various ethnic minority groups in comparison with the majority populations with respect to the general practitioner (GP) services in combination with lower rates for outpatient services [3-5]. Other studies reported either no differences in outpatient care use [6,7] or higher rates among ethnic minorities or immigrant populations [8-12].

Differences in consumption rates could be based upon differences in the incidence of diseases. For some diseases, like diabetes, it has indeed been shown that there are ethnic differences in incidence [13-15]. However, ethnic differences in consumption rates could also have other reasons. For instance, in case of referral of patients by a GP to an outpatient clinic, patients ethnicity might influence the physicians' beliefs about and expectations of patients, and consequently the physicians' actions [16]. There are also indications that, as a result of less effective and satisfying doctor-patient relationship [17], physicians that treat ethnic minority patients are more uncertain in the process of care [18]. Especially in case of language problems, which are common among immigrant populations, the latter might be the case. This could clearly have implications for the referral pattern and the care physicians give. A possible result could be that people from ethnic minorities are more often referred on the basis of vague symptoms, and might therefore less often receive a medical diagnosis. Other possible explanations could be that immigrant patients seek professional medical help more often, not only because they actually do have more health problems, but they also tend to report physical symptoms more often and more expressive. It is suggested that this might be due to the fact that they have a more positive attitude towards care-seeking [19,20].

Since the number of immigrants in the industrialized countries is growing, it is also of growing importance to obtain data on ethnic differences in the use of health care, referral patterns en diagnoses. E.g., in the Netherlands the proportion of non-western immigrants increased from about 3% in 1980, to 6% in 1990 and more than 10% in 2005. A limitation of previous studies is their reliance on self-reports of health care utilization. Although self-reports have been shown to be a valid estimate of health care utilization across socio-economic strata [21], there is less evidence for cross-cultural validity, especially among some of the larger immigrant groups in the western European countries, Turkish and Moroccan people [22]. Ethnic differences in recall bias, non-response and tendency for giving socially desirable answers, could undermine the validity of self-reported measures [23]. For instance, illiteracy and limited proficiency in the native language, both more prevalent among immigrants, will increase non-response rates. Therefore we have chosen for the use of hospital registration data in order to examine ethnic differences in the use of health care.

The ethnic minority populations in the western European countries mainly exist of immigrants who entered the country in the period between 1955–1985 when there was a severe shortage of people to do the unschooled jobs in these countries (first-generation immigrants). In the case that ethnic differences are found, it would also be worthwhile to know whether these differences also persist in the younger generations or whether the consumption rates in younger generations will be more alike those in the majority population.

Using hospital registration data for an outpatient clinic for internal medicine we investigated the following research questions:

(1) Are there differences between ethnic groups in the use of outpatient health services?

(2) Are there differences in reasons for referral and diagnosis between ethnic groups?

## Methods

### Population

From March 2002 to March 2003 the ethnicity of all *new *patients that visited the outpatient clinic of the department of Internal Medicine, Erasmus Medical Center (Erasmus MC), a university hospital in Rotterdam, the Netherlands, was registered (N = 3985). Definition of *new *patients: no referrals in the previous 14 months and referral for medical signs and symptoms that were not examined or treated in an earlier stage. Age of all patients was 15 year and over. This is because children under 15 year do not visit Erasmus MC, they go to a specialised hospital for children.

Based on the next inclusion criteria the study population was selected out of the 3985 patients:

1. Age under 71 year, as the older age groups contained very few immigrants. (N = 3270)

2. The living area, based on zipp codes, is restricted to the municipality of Rotterdam. (N = 1592)

3. Patients ethnicity: Six ethnicities were included: Surinamese, Turkish, Moroccan, Aruban/Antillean, Cape Verdean and Dutch. (N = 1332)

### Data

The research proposal, including the plan of data collection, was authorised by the research ethics committee of the Erasmus MC. We used the country of birth of the patient and both parents to assign ethnicity. We applied the standard definition of ethnicity of Statistics Netherlands and considered a person to be Non-Dutch if at least one parent was born abroad [24]. During the year all new patients were asked for their and their parents' country of birth. Immigrants who were born in the Netherlands and had at least one parent that was born abroad, were considered second-generation immigrants. If a person was born abroad and at least one of the parents did, we defined the person as first-generation immigrant. A six-digit zip code was used for ascribing socio-economic status (based on standardized household income) and based on quintiles determined for Rotterdam [25]. Information about the composition of the population of Rotterdam was obtained from Statistics Rotterdam. A data file was created for the observation period. Persons were allowed to enter the study throughout the study period (open cohort design).

The patient population was divided into two groups: residents from the referral area of the Erasmus MC and patients living in the municipality of Rotterdam. The referral area is part of the municipality of Rotterdam as a whole; the inhabitants of the referral area constituted about 12% of all inhabitants living in the municipality of Rotterdam. The referral area consists of the neighbourhoods surrounding the Erasmus MC, which is for the greater part a deprived area, for which the hospital has a local community service.

Medical reports sent to the general practitioners, when the diagnostic analysis was completed, were scrutinized in order to collect the reason for referral and diagnosis. Both paper medical record and electronic records were used complementary in order to collect the data. Complete data were available for 1070 of 1332 patients. Absence of reports was equally distributed over all ethnic groups. After looking into more detail, 82 patients were not new patients. In total, referral reasons and diagnosis were collected for 988 new patients. Referral reasons were coded according to the International Classification of Primary Care (ICPC) and diagnosis according to the Diagnosis Treatment Combination (DTC). The latter is a system used to finance hospitals in the Netherlands. It is based on formation of groups of patients that have a homogeneous health care use profile. We designed meaningful categories by aggregating ICPC and DTC codes, in order to obtain groups of sufficient size for the analyses. In the appendix the original codes and structure as well as the aggregated categories for ICPC and DTC are presented.

### Contextual information

In the Netherlands general practitioners are the gatekeepers to most other health services. Almost all Dutch inhabitants have a health insurance, at least in the period of this study. That means that there are no financial barriers for seeking professional health care help. In Rotterdam there are, besides Erasmus MC, four more hospitals that offer health care services. However, inhabitants of the referral area mainly visits the Erasmus MC, because of it's local community service for this area.

### Analysis

With regard to research question 1 it was examined whether ethnic minority groups had a higher or lower use of the outpatient clinic than could be expected *from their relative distribution in the population*. In order to estimate rate ratios (Relative Risks) and 95% confidence intervals (CI) of health care use by ethnicity, in research question 1, Poisson regression analyses were carried out. Ethnicity was the independent and use of health care the dependent variable, while adjusting for age, sex and socio-economic status. For the Poisson distribution, the patients constituted the numbers of observed events (numerator). A base group (as a reference) represents the rate (denominator) at which these events occur. The population of the municipality of Rotterdam, including the ethnic distribution of it, constituted this group. For analyses restricted to the referral area, the population of the referral area was the base group. Both base groups were exactly defined grounded on six-digit zip codes. The composition (concerning age, sex and socio-economic status) of the base groups was obtained from Statistics Rotterdam. We used the multiplicative (relative) risk, which is the standard Poisson regression model. The statistical package used was EGRET (version 2.0.1).

With regard to research question 2, we examined whether there were ethnic differences in reasons for referral and diagnosis *within the study population of patients*. For the research questions about ethnic differences in referrals and diagnosis, the reference consisted of the patient group. We did not have the data to estimate odds ratios for the population of Rotterdam, that is why we restricted these analyses to the patient population. For these questions logistic regression was used in SPSS (version 11). Ethnicity was the independent variable and reason for referral respectively diagnosis was the dependent variable (yes/no referred for gastro-intestinal signs and symptoms; yes/no gastro-intestinal diagnosis). We adjusted for age, sex and socio-economic status.

The analyses of research question 1, concerning the population of Rotterdam, were restricted to people aged 15–70, as the older age groups contained very few immigrants. In the models we adjusted for sex, age (10-year age categories), and socio-economic status (SES; quintiles). The analyses concerning differences in generation were restricted to people 15–45, as the second-generation immigrants contained very few people above 45 years.

## Results

In total, 4438 new patients visited the outpatient clinic. From these 4438 patients, ethnicity was registered for 3985 patients (90%). Only residents of the municipality of Rotterdam were included (40%). Six ethnicities were included: Surinamese, Turkish, Moroccan, Aruban/Antillean, Cape Verdean and Dutch (33%). Among the ethnic minorities Surinamese was the largest, and Antillean/Aruban accounted for the smallest group of patients. All patients were referred by their GP, because of the gatekeeper role of the GP in the Netherlands. In table 1 characteristics of the research population are presented. In total 1332 patients remained.

**Table 1:** Population by living area, ethnicity, mean age, sex, generation and socio-economic status. N = 1332.

Referral area	Dutch	Surinamese	Turkish	Moroccan	Antillean Aruban	Cape Verdean	S, T, M, A/A, C Together*
Erasmus MC							
N = 320	124	62	57	36	11	30	196
Mean age	55.0	46.5	41.8	41.4	39.6	50.4	44.4
% men	43.5	32.3	31.6	38.9	63.6	53.3	38.3
% 2^nd ^generation	-	6.5	5.3	5.6	-	-	4.6
							
Municipality	Dutch	Surinamese	Turkish	Moroccan	Antillean/Aruban	Cape Verdean	S, T, M, A/A, C Together*
Rotterdam							

N = 1332	852	174	126	79	50	51	480
Mean age	56.1	45.0	41.4	42.1	41.3	47.3	43.4
% men	40.1	33.9	38.1	44.3	44.0	51.0	39.6
% 2^nd ^generation	-	7.5	8.7	5.1	2.0	3.9	6.5
% lowest SES level	33	62.1	80.2	83.5	54.0	76.5	71.0

* Surinamese, Turkish, Moroccan, Antillean, Aruban and Caper Verdean together

For the referral area of the Erasmus MC, immigrant people have an increased use of the outpatient clinic compared to Dutch people, adjusted for sex and age. The increased use was expressed by relative risks of consultations, which ranged from 1.29 in the Cape Verdean group to 1.82 in the Turkish group. The difference was statistically significant only for Surinamese, Turkish and Moroccan people.

For the municipality of Rotterdam, all immigrant groups included in this study, had a significantly increased use of outpatient care, adjusted for sex and age. Again Turkish immigrants had the highest rates; relative risks ranged from 1.65 in the Antillean/Aruban group to 1.97 in the Turkish group. In table 2A relative risks are presented for all ethnic minorities compared to the native Dutch.

**Table 2:** Relative risks are presented for all ethnic minorities compared to the native Dutch.

A Referrals to Internal Medicine outpatient care by Ethnicity (Relative risks (CI 95%) with Dutch as reference; age 15–70).						
	Surinamese	Turkish	Moroccan	Antillean/Aruban	Cape Verdean	p-value*
						
Referral area Erasmus MC N = 320						
						
Adjusted for sex and age	1.47 (1.05–2.06)	1.82 (1.29–2.56)	1.49 (1.00–2.21)	1.46 (0.78–2.75)	1.29 (0.85–1.97)	0.02
Additional adjustment for socio-economic status	1.49 (1.06–2.07)	1.84 (1.31–2.59)	1.50 (1.01–2.24)	1.49 (0.79–2.80)	1.30 (0.86–1.99)	0.02
						
Municipality of Rotterdam N = 1332						
						
Adjusted for sex and age	1.88 (1.58–2.24)	2.05 (1.68–2.50)	1.88 (1.48–2.39)	1.67 (1.24–2.26)	1.99 (1.49–2.67)	<0.001
Additional adjustment for socio-economic status	1.83 (1.53–2.19)	1.97 (1.59–2.42)	1.79 (1.40–2.29)	1.65 (1.22–2.24)	1.88 (1.40–2.54)	<0.001

B						
Relative risks (CI 95%) for the use of outpatient care						
N = 385						

Comparison				Relative risk^a^		

1^st ^generation immigrants versus Dutch				1.85 (1.51–2.25)		
2^nd ^generation immigrants versus Dutch				1.08 (0.72–1.63)		
2^nd ^generation versus 1^st ^generation immigrants				0.59 (0.39–0.88)		

*p-value of the overall ethnic differences (Wald test).

^a ^age 15–45, adjusted for sex, age and socio-economic status

Additional adjustment for socio-economic status hardly changed the estimates (table 2A). The largest decrease in relative risk was observed among Cape Verdeans in the analyses for the municipality of Rotterdam, from 1.99 to 1.88. In analyses in which the first and second immigrant generations were distinguished (table 2B), no difference in the use of health care were observed between the second-generation and the native Dutch citizens. In both areas the increased use can be predominantly ascribed to the first-generation immigrants.

In table 3 odds ratios are represented for ethnic differences in referral reasons. Compared to Dutch patients, immigrant patients are less likely to be referred to the outpatient care of the Erasmus MC because of reasons in the category 'indefinite, ambiguous signs'. Further analysis showed that the difference in this referral reason is mainly based on two underlying categories; general weakness/tiredness and memory disorder, which both occurred more frequently among Dutch patients (data not shown). Immigrant patients are more likely to be referred because of reasons in the category 'signs & symptoms gastro-intestinal'. Underlying codes in these are generalized/diffuse abdominal pain/cramps, localized abdominal pain and viral hepatitis, of which all three conditions had a higher incidence among immigrant patients. The only exception in this category is rectal bleeding which had a lower incidence among immigrant patients (data not shown). In the patient population were no ethnic differences in the likelihood to be referred because of reasons in the category 'risk for vascular diseases' or the category with remaining referral reasons.

**Table 3:** Ethnic differences in referral reasons. N = 988

	N total	Dutch	Immigrants	Odds ratios ^abc^
Indefinite, ambiguous signs	144	119	25	0.46* (0.27–0.77)
Signs & symptoms gastro-intestinal	298	160	138	1.45* (1.05–2.00)
Risk for vascular diseases	139	88	51	1.11 (0.71–1.71)
Remaining category	407	257	150	0.90 (0.66–1.23)

^a ^Adjusted for sex, age and SES.

^b ^Confidence Interval 95%

^c ^Immigrants versus Dutch as reference.

*p < 0.05

After adjusting for socio-economic status ethnic differences only decreased slightly, indicating that ethnic differences in socio-economic status hardly explained the differences in referral reasons for patients that were referred to the Erasmus MC.

In table 4 odds ratios are represented for the categories of the diagnosis, as made by the internist. With regard to diagnosis, immigrant patients have an increased risk to be diagnosed with 'liver' diseases and they have a lower risk for the category 'analysis without diagnosis'. The dominant code in the category liver is hepatitis B/C. The category 'analysis without diagnosis' constituted a set of underlying codes which all have in common that extensive medical examination took place without giving a pathological diagnosis. The underlying codes discriminate between different complaints, from which general weakness/tiredness and a collection of residue complaints (e.g. impairment of visual acuity, sickness, amnesia) occurred more often among Dutch patients. Analysis of abdominal pain without resulting in a pathological diagnosis on the contrary, occurred more often among immigrant patients.

**Table 4:** Ethnic differences in diagnosis. N = 988

	N total	Dutch	Immigrants	Odds ratios ^abc^
Diagnose category 'risk vascular diseases, including diabetes mellitus'	143	93	52	1.12 (0.72–1.72)
Diagnose category 'Liver diseases'	75	32	43	1.75* (1.00–3.07)
Diagnose category 'Gastro-intestinal'	200	118	82	1.07 (0.74–1.55)
Diagnose category 'Analysis without diagnosis'	278	194	84	0.68* (0.48–0.95)
Diagnose category 'Endocrinology without diabetes mellitus'	108	60	48	0.90 (0.56–1.44)
Remaining category	184	127	55	1.17 (0.76–1.81)

^a ^Adjusted for sex, age and SES.

^b ^Confidence Interval 95%.

^c ^Immigrants versus Dutch as reference.

*p < 0.05.

Ethnic differences in risk for 'liver diseases' are partly explained by differences in socio-economic status; after adjusting for socio-economic status the differences in risk became smaller. For 'liver diseases' the risk decreased from 1.96 to 1.75, but retained a borderline significance. For 'analysis without diagnosis' the risk decreased slightly after adjustment for socio-economic status (from 0.62 to 0.67), it retained statistical significance.

Finally, we also analysed ethnic differences in the risk of getting a certain diagnosis given the referral reason and looked for ethnic differences in this relationship. There appeared to be no differences between the ethnic groups under study, except for the category 'gastro-intestinal signs & symptoms', in which immigrant patients were more likely to receive a diagnosis in the category 'liver' (data not shown).

## Discussion

There is a higher number of new patient referral visits of Surinamese, Turkish and Moroccan immigrants, living in the referral area of the Erasmus MC, compared to native Dutch people than could be expected from their relative distribution in the population. In Rotterdam municipality the five largest ethnic minority groups all demonstrate a higher use of the outpatient care facilities. This increased use can be predominantly ascribed to the first-generation immigrants; second-generation immigrants do not appear to have an increased use of health care services. Immigrant patients who visited the outpatient clinic were more likely to be referred because of 'gastro-intestinal signs & symptoms' and less likely to be referred because of 'diffuse and ambiguous signs'. Regarding ethnic differences in diagnosis, we noted an increased risk of 'liver related diagnosis' and a decreased risk of 'analysis without diagnosis' for immigrant patients.

We have to consider a few limitations of the current study. Although over 4000 new patients were registered in the hospital, numbers for those eligible for the study were small for some ethnic groups, and especially for second-generation immigrants. Therefore, not all research questions could be examined for the ethnic groups separately, nor could the first-generation be distinguished from second-generation immigrant for all research questions. For our second research question it was necessary to aggregate all ethnic groups to one 'immigrant' group. The aggregation was justified by the outcomes of table 2, in which all ethnic groups show a deviated use of health care in the same range and direction. A similar limitation concerns the aggregating of codes of *referral reasons *and *diagnosis*. In the results of research question 2 no ethnic differences were found for the *referral reason *'risk factor vascular disease'. However, looking in more detail shows large differences between the ethnic minority groups for more specific referral reasons. Surinamese and Cape Verdean patients often are referred with the most prevalent underlying risk of vascular diseases, namely hypertension. The same holds with regard to ethnic differences in *diagnosis*: we found no different risk of *diagnosis *'risk factor vascular diseases' regarding ethnicity. But the underlying codes showed that diabetes mellitus was significantly more prevalent among referred immigrant patients and dyslipidemia more common among Dutch patients. Odds ratios have to be interpreted in a relative sense, because they were calculated for the closed group of patients that visit the outpatient clinic of Erasmus MC. An apparent lower odds ratio might be the result of higher rates in other groups of diagnosis.

In the second place ethnicity is based on countries of birth. Although this is a well-accepted definition [6,26-28] we were unable to address ethnic variations within immigrant groups. Differences in the use of health care may have been more differentiated within certain ethnic minority groups, especially for the ethnically diverse Surinamese and Antillean/Aruban population.

We approximated socioeconomic status at the individual level by making use of mean neighbourhood incomes, a variable at the ecological level. This measure may not be equally good for all ethnic groups. In some groups, the place of residence is determined by the mean socioeconomic status of a neighbourhood, whereas in others it is predominantly determined by the ethnic composition of a neighbourhood. In that case, neighbourhood income may be a less valid indicator of socioeconomic status. For Antilleans this does not seem to be the case, however for Turks, Moroccans and Surinamese a somewhat larger proportion (5 to 15%) of the population belonged to the lowest income quintile according to the measure at the ecological level than according to the measure at the individual level. This means that the place of residence of Turks, Moroccan and Surinamese may be more strongly determined by factors other than neighbourhood income. As the discrepancy was fairly small, the influence of the differential validity on the outcomes of this study would be limited [25].

Besides Erasmus MC, there are four more hospitals in Rotterdam that offer health care services. Differences in preference for Erasmus MC could have introduced the differences in health care use. For at least the referral area, this seems hardly the case. A survey among general practitioners in the referral area reported a slightly different referral pattern among different ethnic groups to Erasmus MC and other hospitals in Rotterdam (unpublished data). General practitioners send immigrant patients more often than Dutch patients to the internal medicine outpatient's care of Erasmus MC. The difference is (at most) 5% and cannot explain the increased use of 80% by immigrant patients. Additional support for our assumption that potential differences in referral patterns (due to preferences or the reputation of the Erasmus MC) between ethnic groups in Rotterdam municipality, have had little influence on the outcomes of our study comes from the analysis of the ethnic differences *in referral reasons *for both areas separately (data not shown). The findings at least indicate that there are no ethnic differences in referral reasons between the referral area and Rotterdam as a whole. Herewith a correct inference for the population of Rotterdam municipality is deduced, since the assumption for representativeness of the patient sample seems to be supported.

Remarkable is that the ethnic differences in likelihood of being referred are higher when focussing on Rotterdam municipality than when focussing on the referral area. It is uncertain whether this can be attributed to the prevalence of certain diseases, which require special care. Erasmus MC is also a university hospital and delivers tertiary medical care.

The results of this study regarding the *use of health care *differ from the results of Stronks et al., who reported no differences in the use of outpatient care [6], likewise using registration data. An explanation could be that they addressed outpatient care clinics comprising of several types of specialists, while we made a restriction to internal medicine. Immigrant patients are known to have a higher incidence of several diseases and syndromes, which are referred to the internal medicine clinic (i.e., diabetes, liver diseases and gastro-intestinal complaints). Diseases referred to other outpatient care clinics probably are more equally distributed among different ethnic groups[8]

The results of our study are in agreement with the results of some other studies. Both Manna [29] and Weide and Foets [19] reported an increased risk for immigrant patients for referral with 'signs & symptoms gastro-intestinal'. Some of our results however, differ from the results of other studies. Other studies have reported that reasons for medical consultation among immigrants patient's are more often misunderstood or perceived as not being appropriate by the physician, and that the diagnostic process among immigrant patients might be more complicated because of language barriers, other concepts of disease, and other expressions of pain or other symptoms [30-33] Possibly this could lead to more referrals for indefinite or ambiguous signs and immigrant patients would be more likely to end up in the category 'analyses without diagnosis', but we found the opposite: less immigrant patients came to the outpatient clinic with 'indefinite ambiguous signs' and compared to Dutch patients they have a lower risk for the category 'analysis without diagnosis'. Differences in domains of health care under study may explain the dissimilarity of their results with ours, as these other investigations mainly focussed on general practitioners or on health care in general. Given the Dutch system, where general practitioners are the gatekeepers to most other health services, including the outpatient services, health complaints perceived as inappropriate might have been filtered out by the general practitioner effectively.

A possible explanation for the higher use of outpatient care among immigrants might be a direct reflection of a higher incidence and prevalence of certain diseases. We did not have information about health status, but previous studies have reported a higher incidence of infectious diseases [34], hypertension [35,36], circulatory diseases [37-39], diabetes [40-42], and worse health status in general [8,43] among immigrant groups. Despite these higher incidences, we cannot rule out that referral rates for immigrant groups are inappropriately low or inequitable. Another explanation for higher use could be different styles/patterns in referring immigrant patients and Dutch patients to the outpatient care. Uitewaal [44] reported that more diabetes patients from Turkish descent than native Dutch diabetes patients were referred to the outpatient care. Moreover, immigrant patients asked more for referrals to outpatient clinics, instead of analysis or treatment by the general practitioner[45] It is known that immigrant patients seek professional medical help more often, not only because they actually do have more health problems, but they also tend to report physical symptoms more often. It is suggested that this might be due to the fact that they have a more positive attitude towards care-seeking [20,19] and they have different beliefs concerning health and illness. [46] However we did not find evidence for ethnic differences in mismatch between referral and diagnosis, general practitioners can cause differences in referrals between immigrant and Dutch patients, when communication with immigrant patients is less effective than in consultations with Dutch patients, there is more misunderstanding and also more non-compliance. [47] These explanations could also contribute to the interpretation of the finding that the increased use of health care services predominantly can be ascribed to the first-generation immigrants. Compared to the first-generation, immigrants of the second-generation generally have a higher education, better language skills and have better control of their lives [20]. Thus, second-generation immigrants could become more alike to Dutch patients and their health care use will become more similar. While first-generation immigrants directly benefited from the more favourable socio-economic, public health and health-care conditions in the Netherlands compared with their country of origin, they are not yet affected by the higher risks of diseases associated with prosperity. [25] In the future, next generations immigrants, will be exposed to new risks similar to the risks of the native Dutch. Old risks, like higher risk for infections, will be substituted for risks more comparable to the native Dutch. Concerning first and second generation immigrants, duration of residence might explain the difference in health care use. However, there is no different mean time of residence between first- and second generation of the ethnic groups under study. Most first generation immigrants came to the Netherlands decades ago as labor workers.

Besides the differences in health care use between native Dutch and ethnic minority groups, there also appear to be differences among the ethnic minority groups themselves. Additional analyses showed that Cape Verdean immigrants have a statistically significant lower use of health care than Surinam, Turkish and Moroccan immigrants. Further research is needed to explore why Cape Verdean immigrants are more similar to the native Dutch population regarding health care use.

Because our data are limited to one particular outpatient care unit and moreover to a university hospital, we must be cautious in generalizing the results and the hypotheses generated by this study require further study.

## Conclusion

We conclude that especially first-generation immigrants make significantly more use of the outpatients' care in internal medicine. Ethnic differences might decrease as the share of first-generation immigrants decreases. Concerning this point, it is warranted to monitor the risks of diseases associated with prosperity in the future among immigrant groups. Ethnic differences in referral reasons and diagnosis might be based on a higher prevalence of diseases. Further study is needed to establish this. We found no evidence that the increased use is based on referrals for non-medical reasons, on the contrary. As long as the increased use of outpatient health care is related to ethnic background and the generation of the immigrants rather than to socio-economic status, health professionals have to take ethnicity into account in their daily medical practice. Moreover, they should take the main differences in prevalence of diseases among immigrants into account during the consultations.

## Authors' contributions

LCL drafted the manuscript. and performed the statistical analysis. AHB participated in the design of the study. SWJL and JPM both conceived of the study, and participated in its design. IJ participated in the conceive of the study, participated in its coordination and helped to draft the manuscript. All authors read and approved the final manuscript.

## Appendix

### Aggregated categories of ICPC with underlying codes

#### Referral indefinite, general signs

Weakness, tiredness general, feeling ill, pain general/multiple sites, nausea, feeling anxious/nerves/tense, feeling depressed, feeling/behaving irritable/angry, sleep disturbance, memory disorder

#### Referral signs & symptoms gastro-enterology

Abdominal pain/cramps general, abdominal pain epigastric, heartburn, rectal/anal pain, perianal itching, abdominal pain localized other, dyspepsia/indigestion, flatulence/gas/belching, vomiting, diarrhoea, constipation, haematemesis/vomiting blood, maelena, rectal bleeding, incontinence of bowel, change in faeces/bowel movements, abdominal mass nos, abdominal distension, viral hepatitis, injury digestive system other, congenital anomaly digestive system, oesophagus disease, duodenal ulcer, peptic ulcer other, stomach function disorder, appendicitis, hiatus hernia, abdominal hernia other, diverticular disease, irritable bowel syndrome, chronic enteritis/ulcerative colitis, anal fissure/perianal abscess, liver disease nos, cholecystisis/cholelithiasis, disease digestive system other.

#### Referral risk factor vascular disease

Elevated bloodpressure, hypertension uncomplicated, hypertension complicated, lipid disorder, diabetes insulin dependent, diabetes non-insulin dependent, ischaemic heart disease with angina, acute myocardial infarction, ischaemic heart disease without angina, stroke/cerebrovascular accident, cerebrovascular disease, artherosclerosis/peripheral vascular disease, pulmonary embolism, heart failure.

#### Remaining referrals

All rest codes occurring at the outpatient department of internal medicine.

### Aggregated categories of DTC with underlying codes

#### Diagnosis cardio vascular diseases and risk factor cardio vascular disease, including diabetes

Hypertension, stroke (not specified as haemorrhage or infarction), embolism and thrombosis of arteries, aneurysmas, atherosclerosis peripheral, other arterial disorders, post thrombosis syndrome, ischaemic heart diseases, unstable angina, myocardial infarction, heart failure, dyslipidaemia, riskfactors vascular disease, thrombophilia, diabetes.

#### Diagnosis liver

Diseases of liver: Hepatitis B/C, alcoholic hepatitis, livercirrhosis, liver tumours.

#### Diagnosis gastro-enterology

Gastroenterology

#### Signs and symptoms without diagnosis

Diagnostic procedures generated no diagnosis. All diagnostic procedures in the beginning were based on signs (i.e. pain) or symptoms (e.g. Fever, deviant laboratory results)

#### Diagnosis endocrinology without diabetes mellitus

Endocrine System Diseases, without diabetes mellitus.

#### Remaining diagnosis

White rule remaining diagnosis

## Pre-publication history

The pre-publication history for this paper can be accessed here:


